# Factors associated with the differential distribution of cetaceans linked with deep habitats in the Western Mediterranean Sea

**DOI:** 10.1038/s41598-022-14369-6

**Published:** 2022-07-28

**Authors:** Estefanía Torreblanca, José-Carlos Báez, Raimundo Real, David Macías, Salvador García-Barcelona, Francisco Ferri-Yañez, Juan-Antonio Camiñas

**Affiliations:** 1 Departamento de Biología Animal, Biogeography, Diversity, and Conservation Research Team, Facultad de Ciencias, Universidad de Málaga, Malaga, Spain; 2 Centro Oceanográfico de Málaga, Instituto Español de Oceanografía, Fuengirola, Spain; 3 Instituto Iberoamericano de Desarrollo Sostenible (IIDS), Universidad Autónoma de Chile, Av. Alemania 1090. Temuco 4810101, Región de la Araucanía, Chile; 4 Helmholtz Centre for Environmental Research-UFZ, Halle, Saale Germany; 5 Academia Malagueña de Ciencias, Malaga, Spain; 6 Asociación Herpetológica Española, Madrid, Spain

**Keywords:** Ecology, Zoology

## Abstract

Deep-habitat cetaceans are generally difficult to study, leading to a limited knowledge of their population. This paper assesses the differential distribution patterns of three deep-habitat cetaceans (Sperm whale—*Physeter macrocephalus,* Risso’s dolphin—*Grampus griseus &* Cuvier’s beaked whale—*Ziphius cavirostris*). We used data of 842 opportunistic sightings of cetaceans in the western Mediterranean sea. We inferred environmental and spatio-temporal factors that affect their distribution. Binary logistic regression models were generated to compare the presence of deep-habitat cetaceans with the presence of other cetacean species in the dataset. Then, the favourability function was applied, allowing for comparison between all the models. Sperm whale and Risso’s dolphin presence was differentially favoured by the distance to towns in the eastern part of the western Mediterranean sea. The differential distribution of sperm whale was also influenced by the stability of SST, and that of the Risso’s dolphin by lower mean salinity and higher mean Chlorophyll A concentration. When modelling the three deep-habitat cetaceans (including Cuvier’s beaked whale), the variable distance to towns had a negative influence on the presence of any of them more than it did to other cetaceans, being more favourable far from towns, so this issue should be further investigated.

## Introduction

There is a wide interest in the study of the distribution and habitat of marine mammals, e.g.^1–3^, in part because they present higher conservation concern and threat levels compared to terrestrial mammals^4–6^. Cetaceans have an important top-down control role in the trophic web despite their relatively low biomass^7,8^. Cetacean species are considered sentinels^9^ and indicators^10,11^ of marine ecosystems. However, cetaceans remain generally poorly known^4,10,12^, especially deep diving cetaceans such as the sperm whale (SW-*Physeter macrocephalus),* the Risso’s dolphin (RD-*Grampus griseus*) and the Cuvier’s beaked whale (CBW-*Ziphius cavirostis*). These are primarily teuthophagous species with a similar feeding ecology in deep habitats^13–15^. Many authors point out the need to study and monitor them^3,16–20^ because there is still a lack of knowledge about the population biology and conservation of deep-diving cetaceans. In the western Mediterranean sea, several studies have been conducted in the Pelagos Sanctuary for Mediterranean Marine Mammals^15,18,21–23^ and the Alboran sea^24,25^. Other poorly known important areas can also be found in the rest of the basin^26–29^, such as the submarine canyon of Cuma in the Tyrrhenian sea for sperm whale^30^, the Central Tyrrhenian Sea for Cuvier’s beaked whale^31^ or the Ionian sea for Risso’s dolphin^32^. Therefore, protection for cetaceans beyond the Pelagos Sanctuary is being claimed^25,33,34^.

The sperm whale has a widespread distribution and is globally enlisted as "vulnerable" by the International Union for the Conservation of Nature (IUCN) Red List of Threatened Species^35^. However, the Mediterranean management unit is classified as "endangered", by both the IUCN^36^ and the Agreement on the Conservation of Cetaceans in the Black Sea, Mediterranean sea and Contiguous Atlantic Area (ACCOBAMS)^37^. The Risso’s dolphin is globally enlisted as “least concern” in the IUCN Red List^38^, but in the Mediterranean the IUCN declares this species as “data deficient”^39^. Moreover, this population is in the appendix II of the Convention on the Conservation of Migratory Species of Wild Animals (CMS). The IUCN classifies the Cuvier's beaked whale as “least concern” globally^40^ and “data deficient” in the Mediterranean sea^41^ but, new research on CBW suggests that this assessment can be upgraded^33^.

The population of Mediterranean SW, as proposed by Drouot et al*.*^42^, differs from that of the North Atlantic Ocean and consequently, it should be managed as a different unit^43^. Recently, Lewis et al*.*^44^ estimated that about 1,800 individuals reside in the Mediterranean sea, considering that the population has decreased in the last 40 years^24^. Ship strikes pose a threat since they can cause serious injury and mortality^28,45^. Severe wounds have been detected in the 8% of the total individuals examined in the north-western Mediterranean sea by Alessi et al.^46^. Entanglement in driftnets has been considered the primary cause of mortality for sperm whales in the Mediterranean sea for decades^20^. Since the banning of driftnets in 2002, bycatch rates have decreased but still occur and currently entanglement is considered the second cause of mortality after ship strikes^45^. Underwater noise and pollution are also a matter of concern for this species^20,36^. This includes plastic debris that can be ingested by sperm whales, which has even been speculated to cause death^19^.

Risso’s dolphin distribution is driven by cephalopods, their primary prey^47^, usually at 400–1000 m depth^48^ but also in shallower waters^13^. Population trends cannot be assessed in the Mediterranean due to a lack of data^49^. Risso’s dolphins are found entangled in fishing gears more often than other cetaceans^50^. Pollution is also a concern for their conservation, as they are negatively affected by plastic debris and noise^51–53^.

Cuvier’s beaked whales have one of the highest population densities in the Alboran sea^25^ and its abundance in the Mediterranean sea has been estimated to 5799 individuals (CV = 24%)^33^. This species is threatened by underwater noise, plastics, driftnets^25,53^ and anthropogenic noise, that has also been linked to mass stranding events of this species^41^.

It has been suggested that deep-diving cetaceans in the Mediterranean sea may be competing for food resources, but also it has been hypothesized that coexistence may be facilitated by spatial and/or temporal segregation^15,23^.

Species distribution models (SDMs) are a widely used tool that analyze presence data, e.g.^55^. While systematic sampling is the optimum way to obtain data for modelling species distribution, the economic investment in marine environmental research is insufficient to obtain good spatial and temporal coverage of cetaceans^54^. In this context, opportunistic data is sometimes available in areas not completely covered by other studies and has proven to be useful to increase species distribution knowledge^55–57^. Since 1993, the Spanish Institute of Oceanography (IEO) has been collecting opportunistic sightings of cetaceans in the western Mediterranean sea. Opportunistic sightings (OS) are defined as incidental sightings performed from vessels not necessarily involved in marine observations. We used OS collected by people (either scientific or not) on fishing and recreational boats. This included all the cetacean species recorded during 21 years. This kind of data could provide valuable information about the differential distribution of species since the presence of other cetaceans can be used as contrasting state of the target species, to model in a similar way as Esteban et al*.*^58^.

In this study, we aimed to assess the distribution pattern of three cetacean species linked to deep habitats (SW, RD, CBW), compared to other cetacean species in the western Mediterranean sea, taking advantage of the opportunistic dataset of the IEO. Although it is also a deep-diver, the habitat of the pilot whale (*Globicephala melas*) is linked to coastal and continental areas^59^, therefore we did not include this species in our analysis. We explained the differential distribution in terms of environmental and spatio-temporal patterns inferred using species distribution modelling techniques.

## Results

**Figure 1:**
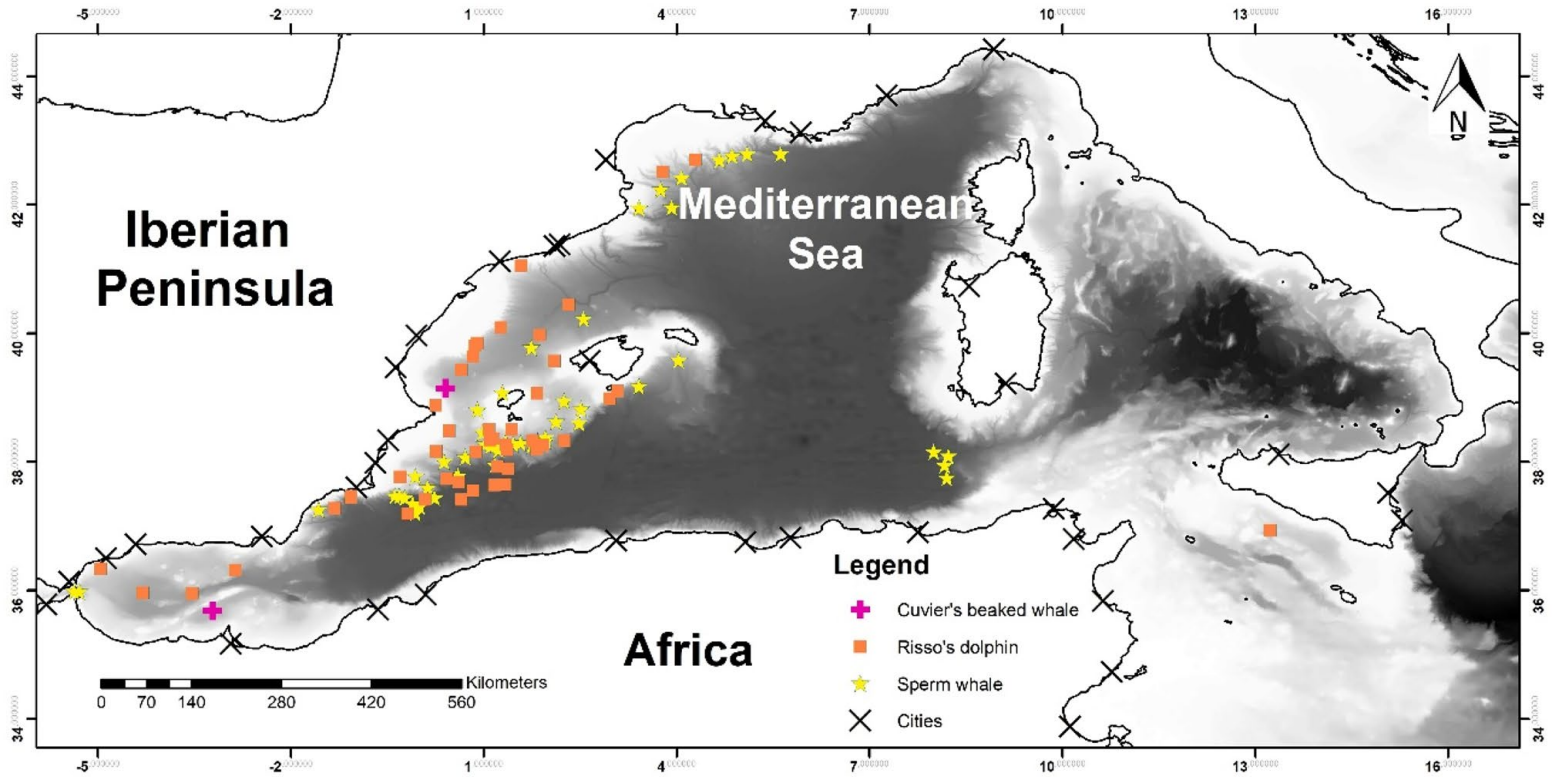
Distribution of the opportunistic sightings (OS) of deep-habitat cetaceans in the dataset. Bathymetry and location of cities with more than 100,000 inhabitants are also displayed. Maps were generated using ArcGIS 10.6 (source: Esri, DigitalGlobe, GeoEye, Earthstar. Geographics, CNES/Airbus, DS, USDO, USGS, AeroGRID, IGN, and the GIS User Community: www.esri.com).

A total of 842 OS of all cetacean species was used in this study (Fig. 1), including 52 OS of sperm whales (SW), 49 of Risso’s dolphin (RD) and 2 of Cuvier’s beaked whale (CBW), but no model was made for CBW. Sperm whale sightings were mostly recorded off the Gulf of Lions, southwest of the Balearic Islands and south of Sicily. Risso’s sightings were also recorded in the southwest of the Balearic Islands but also closer to the Spanish coast and in the Alboran sea. The two beaked whales were observed in the Alboran sea and in the southern Balearic sea.

We obtained several significant explanatory models for the presence of deep-habitat cetaceans according to the two analysed factors. This means that the differential use of the waters by these species with respect to that of the other cetacean species was affected by both the spatio-temporal and the environmental factors.

First (models 1) we modeled presences of SW *versus* presences of the other cetaceans in the dataset. Then (models 2) SW presences were modeled *versus* the presences of the other cetaceans in the dataset but excluding the deep-divers (RD &CBW). In the third set of models (models 3) we tested the opposite as in models 2, that is, SW *versus* the presences of the other species linked with deep habitats (RD & CBW). Following the same process, models 4 and 5 tested the presence of RD *versus* the rest of cetaceans (models 4) and the species linked with deep habitats (models 5). Finally, we tested presences of cetacean species linked with deep habitats *versus* the other cetaceans (models 6).

The significant selected models and the values of each measure used to evaluate the models can be observed in Table 1.

**Table 1:** Significant differential distribution models and their performance measures.

Model	Spatio-temporal	Environmental	Combined
(1) SW vs All	$-3.829 +0.00929*\mathrm{TD}+0.153*\mathrm{Lon}$	$-1.345-7.396*\mathrm{stdevSST}$	$-2.591+0.155*\mathrm{Lon}-6.549*\mathrm{stdevSST}+0.00828*\mathrm{TD}$
H&L	3.872 d.f.8 P = 0.868	9.844 d.f. 8 P = 0.276	3.074 d.f. 8 P = 0.930
Omnibus	21.031 d.f. 2 P < 0.001	14.562 d.f.1 P < 0.001	26.123 d.f. 3 P < 0.001
	AUC = 0.7 R2Naguelkerke = 0.066	AUC = 0.656 R2Naguelkerke = 0.068	AUC = 0.738 R2Naguelkerke = 0.120
(2) SW vs non-deep-habitat cetaceans	$-3.836 +0.010*\mathrm{TD }+0.156*\mathrm{Lon}$	$-1.254 -7.488*\mathrm{stdevSST}$	$-2.618 +0.00916*\mathrm{TD }-6.497*\mathrm{stdevSST }+0.160*\mathrm{Lon}$
H&L	6.393 d.f. 8 P = 0.603	10.529 d.f. 8 P = 0.230	4.155 d.f. 8 P = 0.843
Omnibus	22.661 d.f. 2 P < 0.001	15.016 d.f. 1 P < 0.001	27.567 d.f. 3 P < 1
	AUC = 0.707 R2Naguelkerke = 0.073	AUC = 0.658 R2Naguelkerke = 0.072	AUC = 0.745 R2Naguelkerke = 0.130
(3) SW vs RD and CBW	$-0.201+0.170*\mathrm{Lon}$	$-61.570-9.132*\mathrm{madSST }+1.693*\mathrm{msalt}$	
H&L	4.255 d.f.8 P = 0.833	8.717 d.f. 8 P = 0.368	
Omnibus	4.622 dg1 P = 0.032	13.859 d.f. 2 P = 0.002	
	AUC = 0.630 R2Naguelkerke = 0.059	AUC = 0.743 R2Naguelkerke = 0.238	
(4) RD vs All	$$-3.238+0.00998TD+\left\{\begin{array}{c}-0.863*Spr\\ -0.561*Sum\\ +0.322*Aut\\ +0*Win\end{array}\right.$$	$48.241-1.370*\mathrm{msalt}$	$70.220-2.005*\mathrm{msalt}+0.010*\mathrm{TD}+1.597*\mathrm{ChlA}+0.202*\mathrm{Lon}$
H&L	17.068 d.f. 8 P = 0.029	15.492 d.f. 8 P = 0.050	7.148 d.f. 8 P = 0.521
Omnibus	14.047 d.f. 4 P = 0.007	8.612 d.f. 1 P = 0.003	22.489 d.f. 4 P < 0.001
	AUC = 0.679 R2Naguelkerke = 0.046	AUC = 0.656 R2Naguelkerke = 0.043	AUC = 0.717 R2Naguelkerke = 0.111
(5) RD vs non-deep-habitat cetaceans	$-3.632+0.010*TD$	$46.847-1.330*msalt$	$$55.590-1.591*msalt+0.013*TD+\left\{\begin{array}{c}-0.870*Spr\\ -0.525*Sum\\ +0.757*Aut\\ +0*Win\end{array}\right.$$
H&L	17.443 d.f. 8 P = 0.026	13.626 d.f. 8 P = 0.092	10.835 d.f. 8 P = 0.211
Omnibus	8.751 d.f. 1 P = 0.003	8.123 d.f. 1 P = 0.004	23.145 d.f. 5 P < 0.001
	AUC = 0.616 R2Naguelkerke = 0.030	AUC = 0.651 R2Naguelkerke = 0.042	AUC = 0.738 R2Naguelkerke = 0.117
(6) SW, RD and CBW vs non-deep- habitat cetaceans	$-3.019 +0.011*TD$	$-1.093 -4.246*stdevSST$	$-2.200 +0.010*TD -3.747*stdevSST$
H&L	17.237 d.f. 8 P = 0.028	4.084 d.f. 8 P = 0.849	6.519 d.f.8 P = 0.589
Omnibus	21.262 d.f. 1 P < 0.001	13.070 d.f. 1 P < 0.001	23.690 d.f. 2 P < 0.001
	AUC = 0.637 R2Naguelkerke = 0.048	AUC = 0.604 R2Naguelkerke = 0.044	AUC = 0.669 R2Naguelkerke = 0.079

The SW spatio-temporal model 1 had two variables, geographical longitude (Lon) and town distance (TD). This model showed a higher probability of SW sightings in the eastern part of the studied area, which mainly reflected the scarcity of OS data of this species in the Alboran sea and the Gulf of Cadiz (Fig. 1, Table 1). It also showed that the probability of an OS corresponding to a SW was lower near cities with more than 10,000 inhabitants.

The environmental model 1 included only the Standard deviation of Sea Surface Temperature (stdevSST). As the coefficient was negative, SW were differentially sighted in areas with low variability in SST (Table 1).

The significant explanatory variables for the model 1 with all variables were, Lon, TD and stdevSST (Table 1). Thus, this model indicated that the probability that an OS corresponded to a sperm whale was higher in eastern locations, far from towns and with a lower SST variability.

When modelling SW versus non-deep-habitat cetacean species (model 2), the significant variables for the spatio-temporal model were TD and Lon, while stdevSST was significant for the environmental model. These three variables remained significant for the model with the combination of all variables.

When modelling SW versus RD and CBW (model 3) the significant spatio-temporal model included only the variable Lon, while madSST and msalt were in both the environmental and combined model.

When modelling RD versus the other cetaceans (model 4), the significant variables were TD and Season for the spatio-temporal model, which indicated that the sighting of RD was differentially more probable in Autumn. In the environmental model msalt was the significant variable included. The model with the combination of all variables included msalt, TD, Chlorophyll A and Lon.

When modelling RD versus non-deep-habitat cetaceans (model 5), the significant variables for the spatio-temporal model was TD, while msalt was significant for the environmental model. For the combined model, the significant variables were TD, season and msalt, therefore the variable season became significant when msalt and TD were already included in the model.

Finally, when modelling the three species linked with deep habitats together in contrast with the rest of cetaceans (model 6), the significant variables were TD for the spatio-temporal factor, stdevSST for the environmental factor and both for the combined model.

Two variables that differentially affected SW and RD are salinity and SST. The probability of sighting a SW was higher than that of a RD at lower madSST and higher salinity.

AUC values of the models ranged from 0.604 the lowest to 0.745, being the combined model 2 that with the highest discriminant capacity.

**Figure 2:**
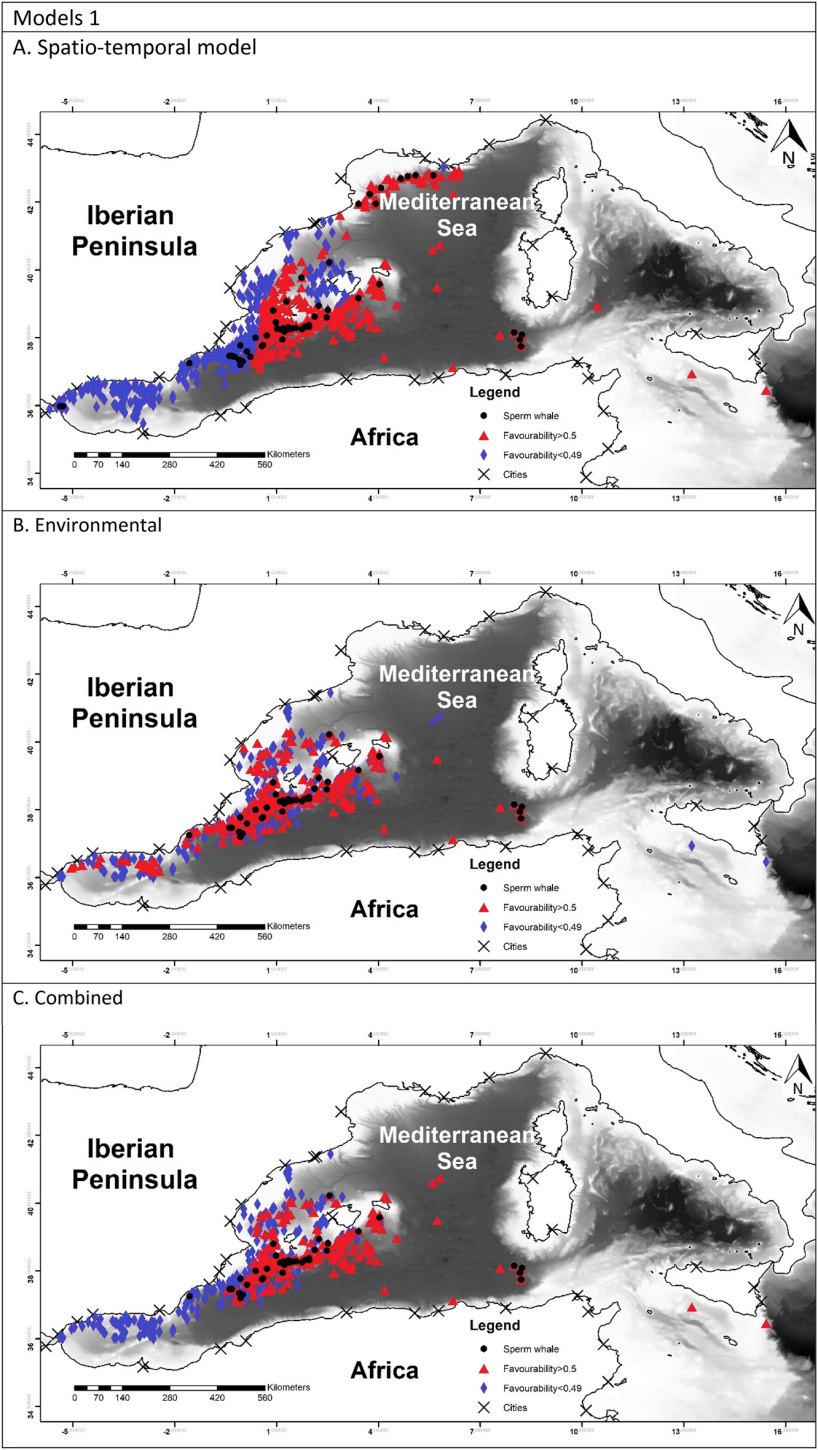

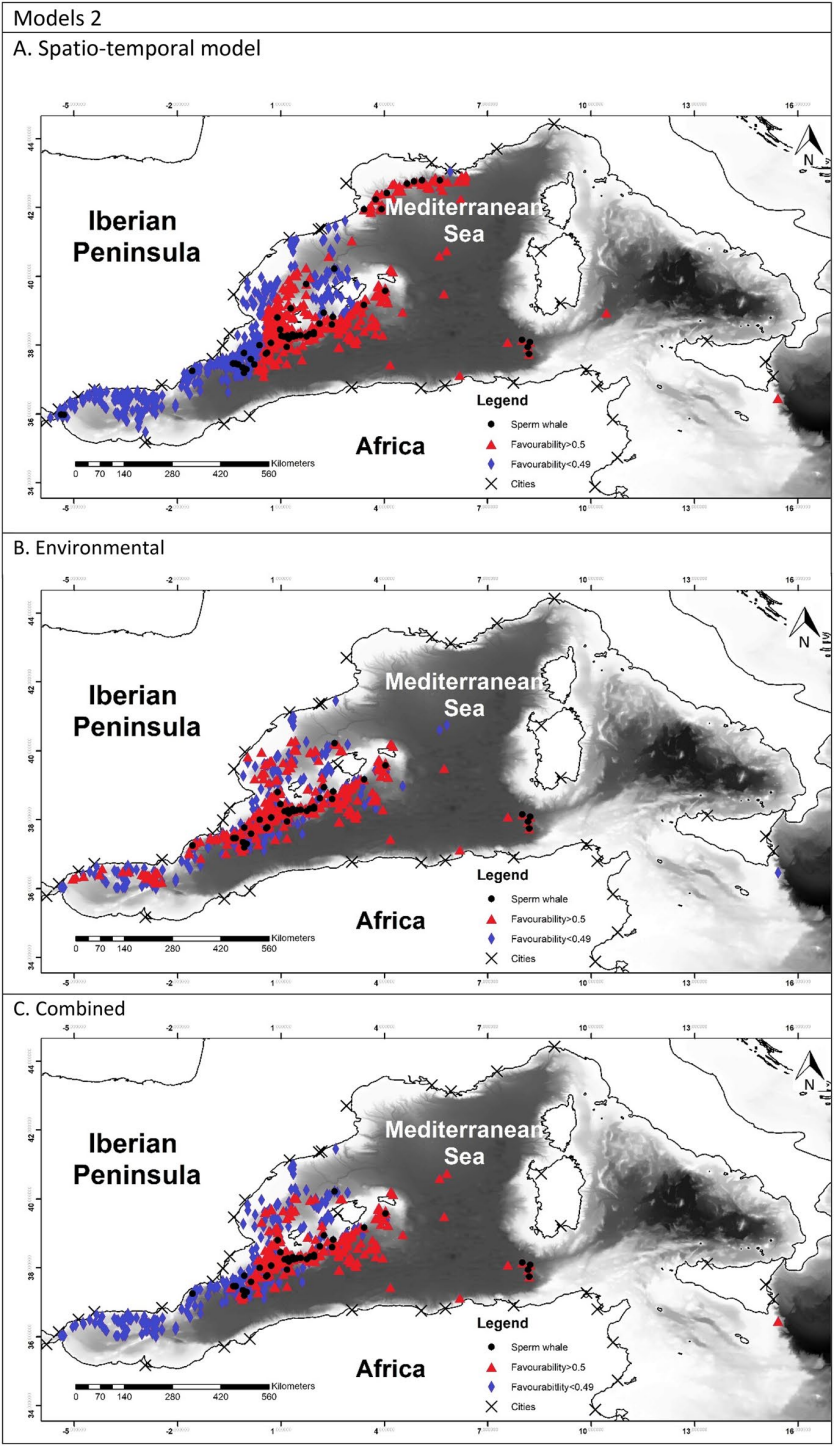

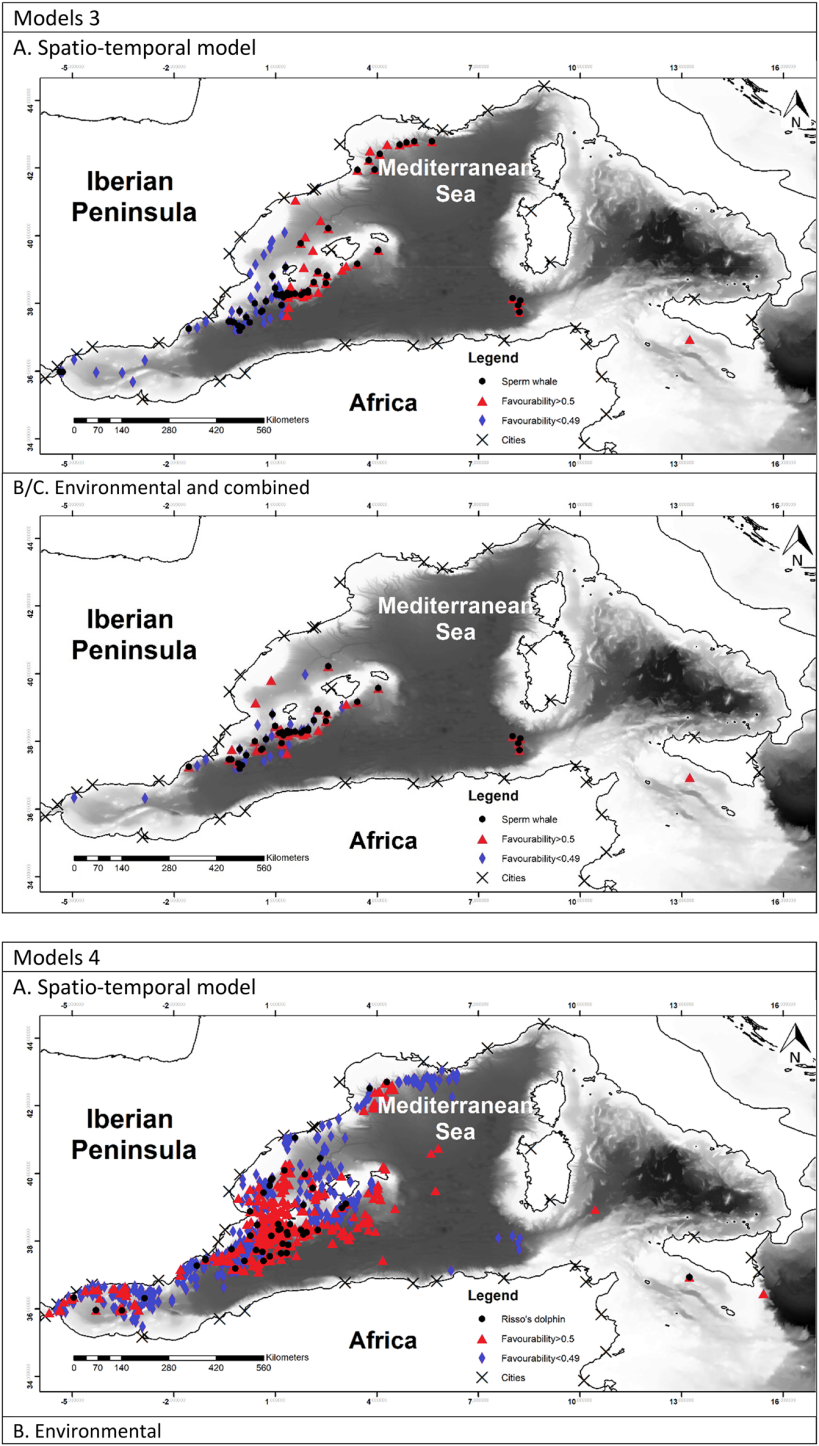

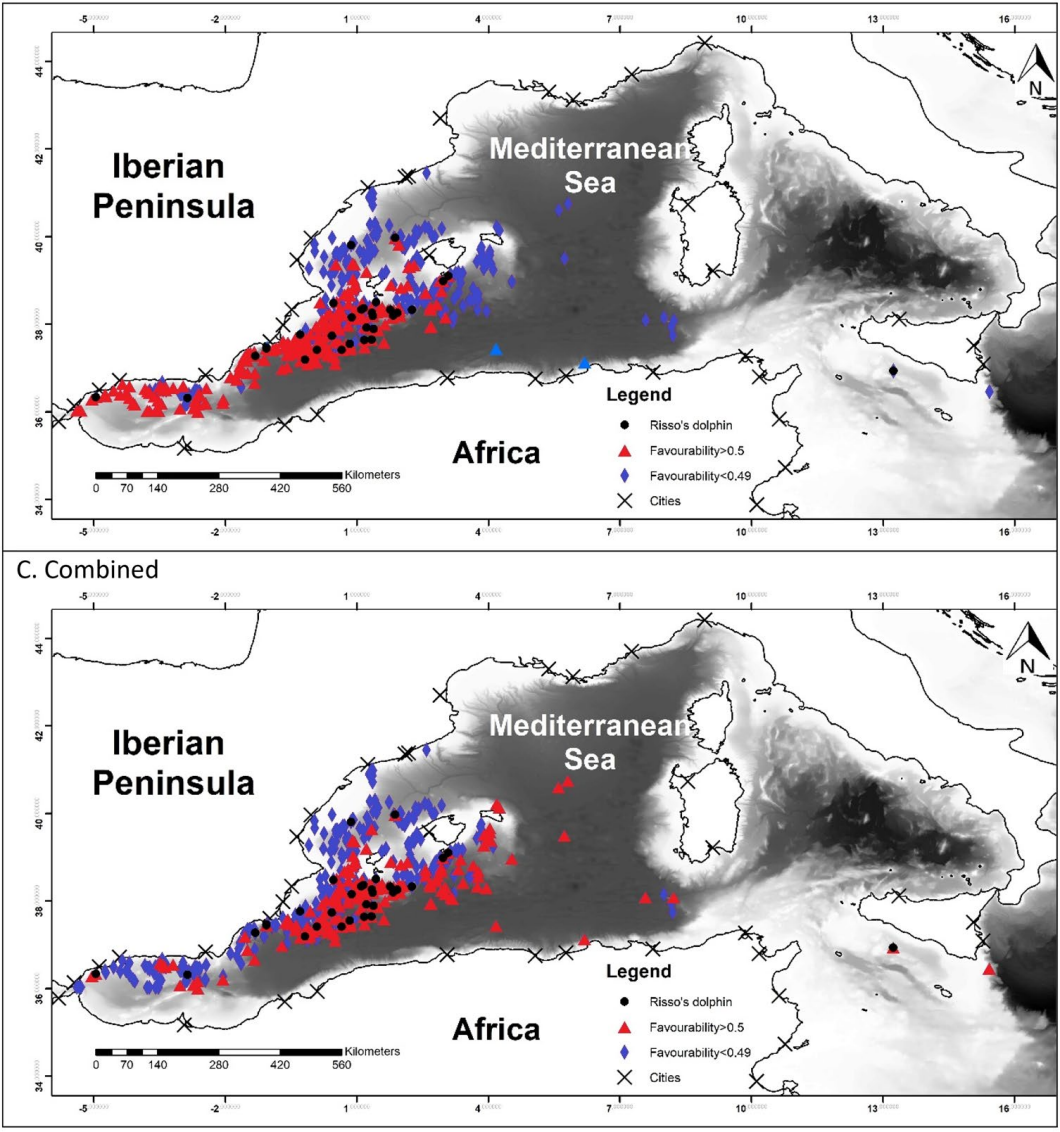

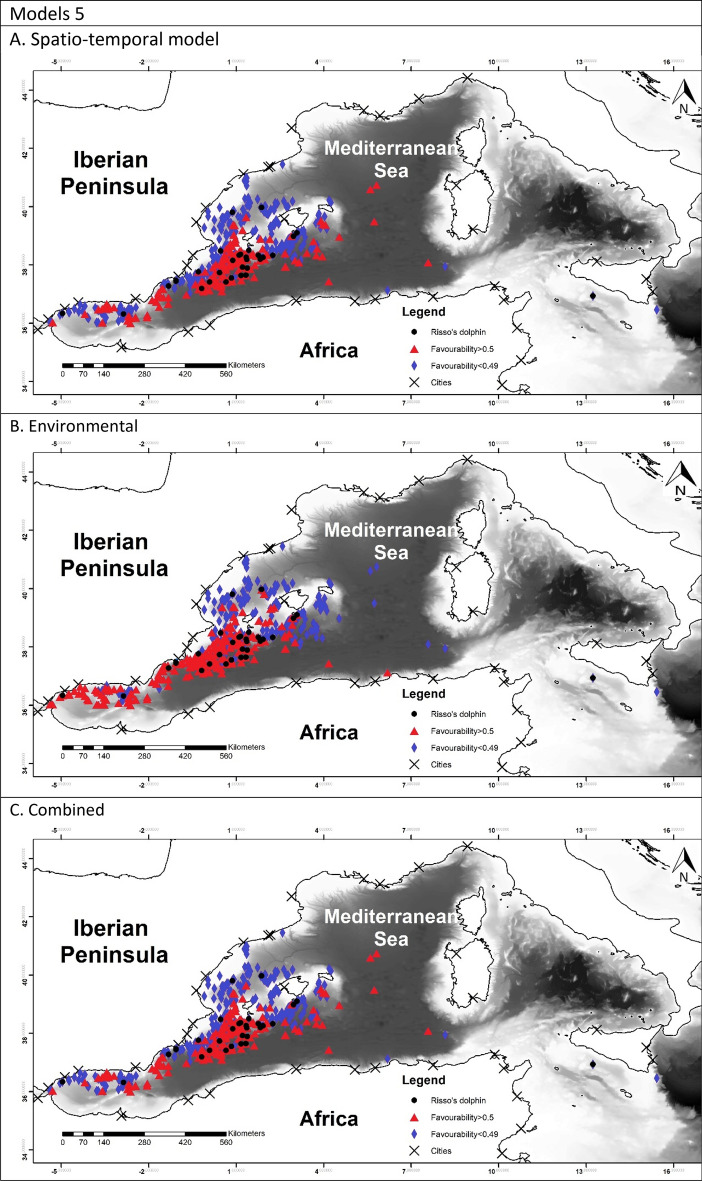

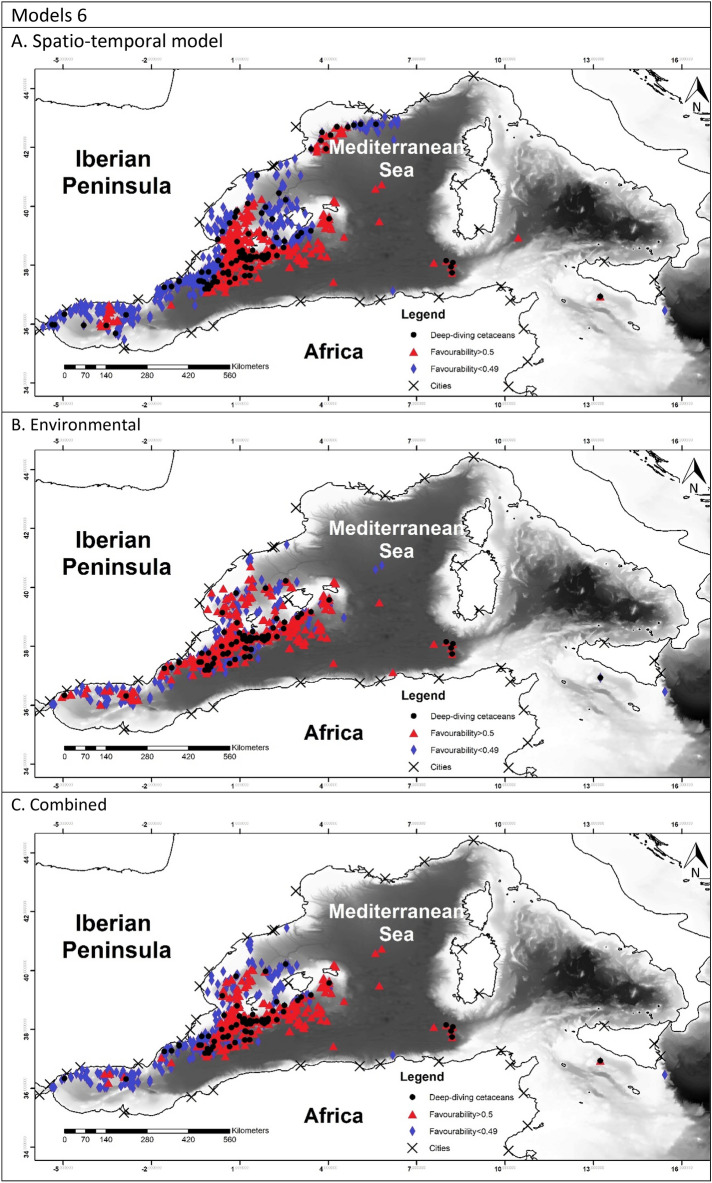
Maps representing the favourability calculated for each model. Black dots represent the presences of the species modelled. Blue triangles represent the locations with the favourability higher than 0.5 and red diamonds represent favourability lower than 0.5. Maps were generated using ArcGIS 10.6 (source: Esri, DigitalGlobe, GeoEye, Earthstar. Geographics, CNES/Airbus, DS, USDO, USGS, AeroGRID, IGN, and the GIS User Community: www.esri.com).

To allow for comparison between the different models, favourability values of each model have been represented in Fig. 2^60^.

The favourability of each model was represented in Fig. 2. In model 1A it is possible to observe a higher favourability in the east, which reflected the effect of TD and Lon. Model 1B showed the favourable areas according to the environmental factor. Finally model 1C represented a more precise favourability area. Models 2 were very similar to model 1A, which can be observed in the maps. Figure 2, models 3 represented the favourability of SW versus RD and CBW, therefore representing the differential favourability between the deep-habitat cetaceans. It could be appreciated the different favourability values of SW and RD, being SW favoured to the east than RD.

## Discussion

The modelling approach used here provided biogeographical information because the influence of the factors was analysed from a macroecological perspective. The habitat preferences obtained with this approach were no absolute, that is, the right interpretation of the results should take in account that the models reflect the differential distribution of the target species in contrast to the presence of the other cetaceans^56^. The probability that a sighting corresponded to a cetacean species linked with deep habitats did not reach 0.5 in the models. This means that, given that a cetacean has been seen, the fact that the sighting belongs to a sperm whale (SW), a Risso’s dolphin (RD) or a Cuvier’s beaked whale (CBW) is a very low probability event.

Statistical models such as those used in this study may be explanatory or predictive^61^. Although predictions could only be developed with a high-quality dataset, Real et al*.*^62^ argued that an uncertainty analogous to that of quantum objects affected the predictability of species distribution, even when all relevant factors were taken into consideration. This was evident when observing cetaceans. Given the nature of this dataset and the uncertainty inherent to species distribution in the marine environment, the models generated here should be considered more explanatory than predictive. In fact, the cryptic nature of these species (sperm whales only spend around 15% of the time at the sea surface^63^) may affect their probability of detection and hamper research efforts^64^ when relaying on OS. This, together with the fact that the sighting of a cetacean is also a low probability event^65^, implied that the data used here were very valuable for research purposes due to the time frame and spatial coverage. According to Azzellino et al*.*^18^, in the northwestern Mediterranean sea (along Ligurian–Provencal coast, the Northwestern Corsican and Sardinian coasts), sperm whales and Risso’s dolphins presences were found mostly associated to the upper slope area, encompassed between the 500 m and 1000 m depth.

Several studies have shown the influence of seasonality on marine mammal sightings. For example, Bănaru et al*.*^8^ detected more sightings of marine mammals in the Gulf of Lion in summer. In the Ligurian sea, although SW occurred throughout the year, it had been reported with higher abundance from August to October^21^ and also higher abundance in summer and autumn in the Sardinian-Balearic sector^31^. Our results suggested that the sighting of RD was differentially more probable in Autumn (models 4 and 5, Table 1), while SW did not differ from other deep-habitat species in its seasonal pattern.

The models 6 (Table 1) suggested a negative anthropogenic effect on deep-habitat cetaceans, given that sighting probability increased as distance to cities increased. This differential effect was maintained in all the models (Table 1). Mussi et al*.*^30^ found that SW sightings increased with distance to the coast, suggesting that SW could be avoiding human activities and that this could result in a reduction of their habitat. Our results corroborated this conclusion, because distance to towns had more explanatory power than distance to the coast or other human disturbance variable such as distance to the main shipping routes.

The models 1, 2 and 4 (Table 1) also indicated that the probability that an OS corresponded to a SW or RD varied with longitude, increasing towards the East, that is, far from the Strait of Gibraltar. Cañadas et al*.*^24^ found a similar pattern for RD, being mainly confined in the eastern part of the Alboran sea. The nearly total absence of sightings of SW in the Alboran sea was in accordance with the observation made by Gannier et al*.*^16^. They found the Alboran sea to be the least frequented area by SW in the Mediterranean sea. This was remarkable, as the Alboran sea is the transition zone from the Atlantic Ocean to the Mediterranean sea and the migrating pathway between Atlantic Ocean and the Mediterranean, with a high diversity and encounter rate of cetaceans^64,66^. We hypothesize that SW, in their migrating path through the Alboran sea, dive deeper and/or closer to the Moroccan coast, avoiding highly populated areas, and are therefore less detectable. This longitudinal pattern was also due, to a lesser extent, to an increase of SW in the easternmost zones of the study area (Fig. 1; Table 1). While in the Strait of Sicily the density of SW seemed to be low^26^, Aïssi et al*.*^34^ predicted a high probability of SW occurrence east of Sardinia and Corsica islands.

In general, it seemed that SW prefer lower temperatures^29^. Praca and Gannier^14^ associated the ecological niche of the SW to a lower SST and salinity. In addition, they found that a high Chlorophyll A concentration was positively associated to the distribution of both SW and RD, although this study was performed in the northwestern Mediterranean sea. Our results, on the contrary, suggested for RD a differential preference for lower salinity and higher Chlorophyll A than SW (Table 1, Model 4), so this pattern may reflect a different factor.

Our results suggested that the stability of SST caused a differential pattern in the SW (Table 1, Model 1). Although Gannier and Praca^17^ found that thermal fronts, especially the North Balearic front, seemed to favour the SW presence in summer^21^, the environmental model and the model combining the two factors (Table 1) showed a lower probability of SW sightings when SST variability was high. Deep water upwelling causes, among other driving factors, the variability of SST. The differential pattern detected in the models reflected upwellings as a feeding ground used by other cetaceans rather than by SW. In other words, we hypothesize that, since SW feed at very deep depths, surface conditions would affect them minimally when compared with cetaceans more directly favoured by upwelling areas. Therefore, it would be interesting to obtain those variables, such as deep currents or temperature for future studies.

A factor known to influence the distribution of SW and RD were seabed topographic variables, commonly used to model the distribution as an indicator of prey availability^16,23,29,66,67^. Depth was included as a potential variable in the spatio-temporal factor analysis^34^, but it was not significant in any of the multivariate models^14^. This is probably due to the higher effect of distance to towns (which was correlated to depth) given that the stepwise procedure prevents the inclusion in the model of variables that were not able to explain the residuals not already explained by more significant variables. This non-relation with depth had been previously detected for RD in the Gulf of Taranto^32^. On the contrary, TD was an important variable in the differential distribution of cetacean species linked with deep habitats.

We have presented several significant models in which variables were first divided in two subcategories and then, all variables were modelled together. This perspective showed that some significant variables, such as distance to towns, could go unnoticed when more variables were considered, even if their explanatory value was important^56^. Model 2 (Table 1), that contrasted SW and the rest of non-deep-habitat cetaceans, showed the best AUC (0.745), which was in the range of other studies, such as Praca et al.^63^, that obtained AUC values of 0.58 and 0.79. Therefore, OS provided valuable information regarding the factors that affected differential distribution, especially when there is a lack of specific surveys^68^. Thus, we encourage organizations to continue recording this kind of data.

The main disadvantage of OS datasets was that they take advantage of sea trips with other purposes to gather information about sightings, and there could be a bias since most of them came from fishing areas. However, this bias was homogeneous also due the long temporal scale of the dataset and could not be the cause of the significant differential patterns detected here. We conclude that deep-habitat cetaceans in the western Mediterranean sea are differentially affected by Lon and TD, compared with other cetacean species, and that opportunistic sighting data can improve previous knowledge of species distribution. We have identified anthropogenic factors that have an important role in the differential distribution of these species and, to delve into this issue, we recommend including anthropogenic variables in future studies.

## Methods

### Study area and data collection

**Figure 3:**
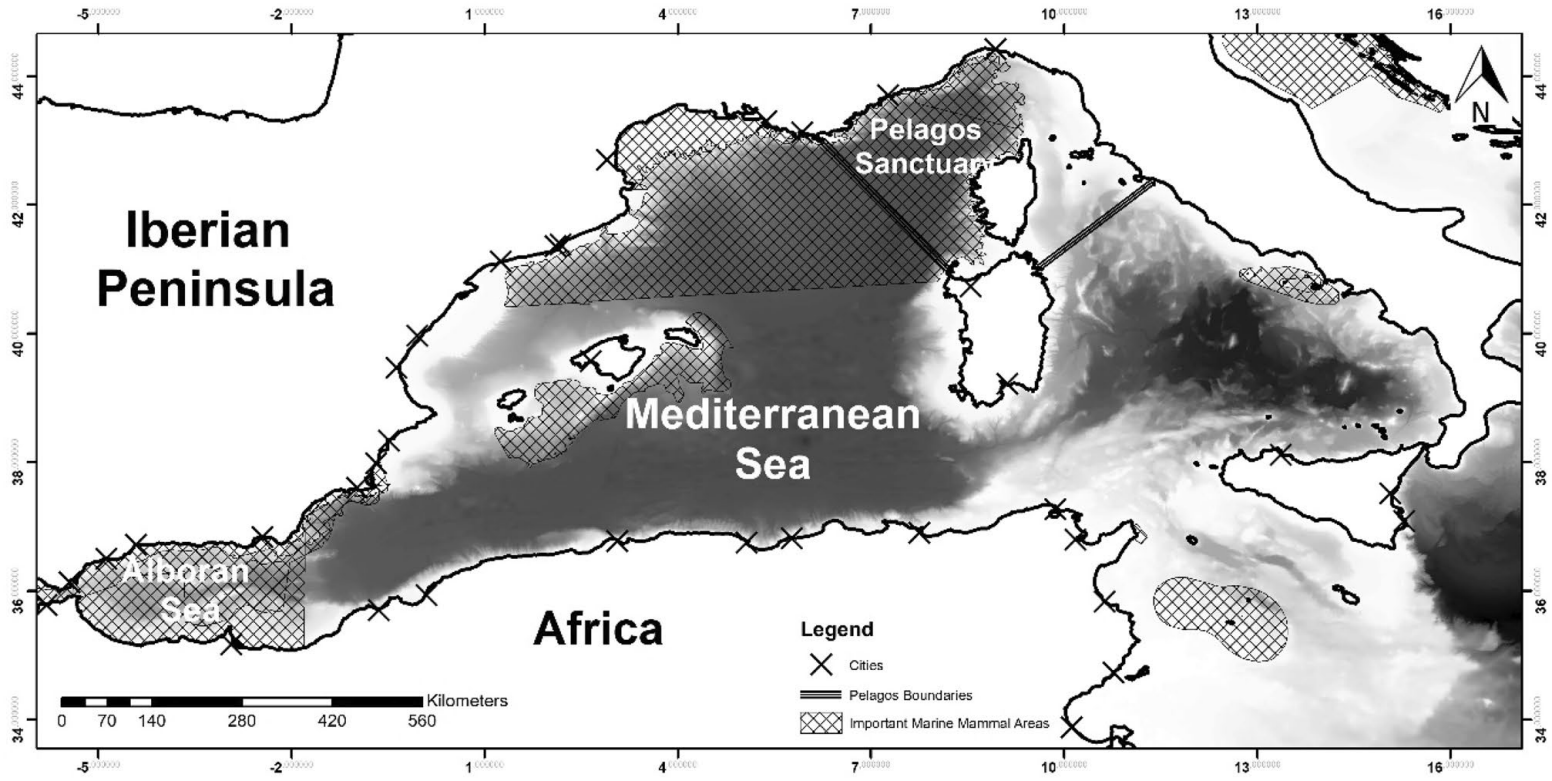
Study area and location of the main cities and Important Marine Mammal Areas. Maps were generated using ArcGIS 10.6 (source: Esri, DigitalGlobe, GeoEye, Earthstar. Geographics, CNES/Airbus, DS, USDO, USGS, AeroGRID, IGN, and the GIS User Community: www.esri.com).

The Mediterranean sea is connected to the Atlantic Ocean through the Strait of Gibraltar and, thus, the waters surrounding this strait constitute an obligated pathway for species migrating between both seas. The study area (Fig. 3) comprised the western Mediterranean sea (35°–43° N; 6° W–15.5° E). Recently, several important marine mammal areas (IMMAs) have been designated by the IUCN in this area (IUCN-MMPATF (2017). The IUCN Global Dataset of Important Marine Mammal Areas (IUCN-IMMA, IUCN Joint SSC/WCPA Marine Mammal Protected Areas Task Force and accessible via the IMMA e-Atlas http://www.marinemammalhabitat.org/imma-eatlas) highlights the importance of this area for cetacean species.

Many authors have proposed to use opportunistic sightings (OS)^56^ collected from different sources (i.e. type of vessels) with other research purposes, to study species distribution and abundance. With a relative low-cost of the data acquisition, opportunistic data can provide relevant information^2,56,67^. We used OS performed by people (either scientific or not) that sail frequently. Such OS data had no effort measures associated with them, given that those sightings were not obtained by a dedicated sampling scheme^56^. Our data was obtained by scientific observers or volunteers with a good knowledge of cetaceans. Moreover, according to our experience training volunteers and professional scientific observers, a deep-diving cetacean is, in general, very easy to identify when compared with the rest of cetaceans inhabiting the western Mediterranean sea. We used a database consisting of 842 of OS of 9 species of cetaceans recorded from 1993 to 2014 by the Spanish Institute of Oceanography (Table 2).

**Table 2:** Number of cetaceans recorded from 1993 to 2014 by the Spanish Institute of Oceanography.

Species	Opportunistic sightings	
**Deep-habitat cetaceans**		
*Physeter macrocephalus*	52	103
*Ziphius cavirostris*	2	
*Grampus griseus*	49	
**Non-deep-habitat cetaceans**		
*Stenella coeruleoalba*	329	739
*Delphinus delphis*	128	
*Tursiops truncatus*	42	
*Globicephala melas*	96	
*Balaenoptera physalus*	70	
*Orcinus orca*	2	
Dolphins	72	
Total	842	

Variables in the logits are ordered according to the order of entrance in the stepwise modelling procedure. *Spr* SPRING, *Sum* summer, *Aut* autumn, *Win* winter, *Lon* geographic longitude, *TD* distance to towns, *stdevSST* standard deviation of sea surface temperature, *madSST* median absolute deviation of temperature, *msalt* salinity, *ChlA* Chlorophyll A concentration, *H&L* Hosmer & Lemeshow test, *AIC* Akaike Information Criterion, *AUC* area under the receiver operating characteristic (ROC) curve. The variables are ordered by punctuation.

### Variables

Each OS record included species identification, date, position, type of vessel, and observer’s name. We related the time and location of each sighting with variables that are believed to affect the distribution of marine species^34,66^. In the literature, environmental and spatio-temporal factors have been widely analysed when generating distribution models for cetaceans^16,66,67^. We considered two sets of variables: spatio-temporal and environmental variables.

#### Spatio-temporal factor

The spatio-temporal factor included the spatial variables Longitude, Latitude, Depth, Coast distance, Main shipping route distance and Distance to towns, and the temporal variable: season (Spring, Summer, Autumn and Winter).

##### Longitude and latitude in degrees (Lon, Lat)

It is obtained during the sighting and shows the longitudinal and latitudinal position. This allowed to consider any spatial autocorrelation in the distribution of the species.

##### Depth (D)

This is a continuous spatial variable that measures the depth of the water column in a chosen point. The bathymetry layer was obtained from the European Marine Observation and Data Network “EMODnet” (http://www.emodnet.eu/bathymetry).

##### Coast distance (CD)

Measures the effect of emerged areas in the sea. The greater the distance, the lesser the influence of the continent and the bigger the predominance of the pelagic ecosystem. We used the layer coastline from the webpage http://www.naturalearthdata.com/downloads/10m-physical-vectors/10m-coastline/, and the tool *Near* of *ArcGIS 10.1*. to calculate the distance in kilometers.

##### Main shipping route distance (MSRD)

It assesses if large commercial vessels affect the distribution of cetaceans, which could be due to the increase of the noise level and/or of the risk of collision with boats. We generated a layer from google maps (https://www.google.es/maps/), with the main routes of the research area. The distance in kilometers from OS to the main route has been obtained with *ArcGis 10.1.*

##### Distance to towns (TD)

Distance to coastal towns with more than 100,000 inhabitants. It was used with the aim of testing if large towns influence the probability of OS. A layer with cities with more than 100,000 inhabitants in the study area was created and the distance in kilometers from OS to the nearest large city was calculated with *ArcGIS 10.1.* Large towns imply debris, pollution, and a major number of sport boards next to the coast.

##### Season

Given that seasonality can affect resident species and the migrant behavior of these species, the seasonal effect on the distribution of sightings was tested. The categorical states of this variable were Spring (*Spr,* 21st March to 20th of June); Summer (*Sum,* 21st June to 22nd September); Autumn (*Aut,* 23rd September to 21st December); Winter (*Win,* 22nd December to 20th March).

#### Environmental factor

The environmental factor was represented with the variables Sea Surface Temperature (SST), Standard deviation of SST, Median Absolute Deviation of SST, Salinity, Water current velocity, and Chlorophyll A concentration.

We downloaded Sea Surface Temperature (SST), Chlorophyll A concentration (ChlA), Salinity, and water current velocity (zonal, meridian, as well as vertical) for each location and date from the NOAA ERDDAP server (https://coastwatch.pfeg.noaa.gov/erddap/). Sea Surface Temperature data was obtained from the AVHRR Pathfinder Version 5.3 (PFV53, https://data.nodc.noaa.gov/cgi-bin/iso?id=gov.noaa.nodc:AVHRR_Pathfinder-NCEI-L3C-v5.3). L3C Sea Surface Temperature dataset has a spatial resolution of 4 km and spans from 1981 to the present. The monthly composite of the daylight measurements was used in this study. We estimated Mean SST value (SST), Median Absolute Deviation of SST (madsst), and Standard Deviation of SST (stdev) for a search radius of 0.2 decimal degrees. Chlorophyll A concentration was obtained from two sources: first as a monthly composite of NASA´s SeaWiFS dataset. This dataset is generated from the Sea-viewing Wide Field-of-View Sensor (SeaWiFS), an eight-channel ocean color sensor on GeoEye's Orbview-2 satellite. Chlorophyll A concentration is computed from the normalized water-leaving radiances using the NASA/GSFC SeaWiFS Project OC4v4 algorithm, e.g.,^69^. The second Chlorophyll A concentration dataset was obtained as a monthly composite of Level 3, Standard Mapped Image at 4 km spatial resolution from the Moderate Resolution Imaging Spectroradiometer (MODIS) on board of the satellite Aqua (product name: L3SMI). We used two sources of Chlorophyll A concentration in order to include all the study period. Salinity and Ocean currents were obtained from the Simple Ocean Data Assimilation ocean/sea ice reanalysis (SODA) dataset version 3.3.1. This is a reconstruction of historical physical parameters of the ocean since the beginning of the twentieth century with a resolution that is chosen to match available data. SODA 3 uses a GFDL MOM5/SIS1 model with eddy allowing for a 1/4° × 1/4° and 50 level resolution (28 km at the Equator down to < 10 km at polar latitudes). SODA3 is described in Carton, Chepurin, and Chen^70^ (2018). All the data were downloaded from the ERDDAP web service from NOAA (https://coastwatch.pfeg.noaa.gov/erddap/) using the *R package “rerddapXtracto”* version 0.3.5.900 (https://github.com/rmendels/rerddapXtracto).

### Statistical analysis

The OS data obtained were transformed into binary data (referred to presence of a species linked with deep habitats versus presence of other cetacean species). Therefore, General Linear Models can be applied as the presence of other species becomes the contrast state of the target species presence^56,71^. That is, we used the presences of other cetaceans, which was termed as “pseudo-absence” by Esteban et al*.*^58^, as the contrasting state of deep-habitat species for modelling purposes. This produced several target binary variables that we have analysed this dataset with binary logistic regression^72^, a binomial method common to study cetacean-habitat relationships. Forward–backward stepwise logistic regression was performed to test if a combination of environmental and spatio-temporal factors could account for the differential distribution of the species. In this procedure, a variable collinearly related to variables already included in previous steps is not accepted unless it is statistically able to explain the residuals not explained in previous steps^72^. This avoids redundancy due to collinearity.

Therefore, several models were obtained when comparing the distribution of the different cetaceans linked with deep habitats with the distribution of other species (Table 3).

**Table 3:** Binary variables and models generated when the presence of species in the first row are contrasted with the presence of species in the first column.

Contrasting data	Sperm whale	Risso’s dolphin	Deep-habitat cetaceans
Rest of cetaceans	Models 1	Models 4	Models 6
Non-deep-habitat cetaceans	Models 2	Models 5	
Risso’s dolphin and Cuvier’s beaked whale	Models 3		

For each binary variable of cetaceans (Table 3), three models were obtained, one with the spatio-temporal variables, another with the environmental variables, and the last one with all variables combined. The Omnibus test^73^ was used to test the statistical significance of the models, where significant differences between − 2LL (minus twice the natural logarithm of the likelihood) of the initial step and − 2LL of the model are evaluated by means of χ^2^ with a degree of freedom, comparing the model in step n with step n + 1^73^. The Wald statistics test was used to test the statistical significance of the individual regression coefficients^74^. The software *IBM SPSS Statistics 25* (IBM, Armonk, NY, USA) was used to perform all statistical analyses. Finally, in order to allow for comparison between the models differing in prevalence, we applied the favourability function^60,75^. Favourability is calculated from the probability value obtained using the following function:

$$F= [P/(1-P)]/[(n1/n0)+(P/[1-P])]$$

where n_1_ is the number of sightings of the target species, n_0_ is the number of sightings of either of the contrasting species and P is the probability value obtained in the logistic regression. The values obtained range from 0 to 1, being 0.5 when the probability for the occurrence of the species is the same as its prevalence in the dataset.

### Model evaluation

Evaluation of these models should consider statistical accuracy as well as ecological significance^14^. Therefore, the goodness-of-fit of the models were tested with the Hosmer and Lemeshow test and the likelihood ratio test^74^. The Hosmer and Lemeshow test compares observed frequencies and expected frequencies of deep-habitat cetacean sightings along the probability gradient, thus assessing the global adjustment of the model. A well fitted model should not have significant differences between the observed and the expected frequency distribution^74^. The likelihood ratio test assesses the models and the best model is the one that maximizes the likelihood function. The discrimination capacity of the model was assessed by the area under the receiver operating characteristic (ROC) curve (AUC)^76,77^. Finally, we calculated the Akaike Information Criterion (AIC) to assess their parsimony^78^. The AIC compares the quality of the models among them and chooses the best^78^.

## Supplementary Information

Supplementary Information.
